# Overexpression of MYCT1 Inhibits Proliferation and Induces Apoptosis in Human Acute Myeloid Leukemia HL-60 and KG-1a Cells *in vitro* and *in vivo*

**DOI:** 10.3389/fphar.2018.01045

**Published:** 2018-09-18

**Authors:** Shuang Fu, Yu Fu, Fang Chen, Yanping Hu, Bi Quan, Jihong Zhang

**Affiliations:** Hematology Laboratory, Shengjing Hospital of China Medical University, Shenyang, China

**Keywords:** acute myeloid leukemia, MYC target 1, cell cycle arrest, proliferation, apoptosis

## Abstract

MYC target 1 (MYCT1), a direct target gene of c-Myc, is a novel candidate tumor suppressor gene first cloned from laryngeal squamous cell carcinoma. The downregulation of MYCT1 has been reported to be associated with carcinogenesis. However, the role of MYCT1 in the development and progress of acute myeloid leukemia (AML) remains unknown and requires further investigation. In this study, we first found that the expression level of MYCT1 was significantly lower in the bone marrow (BM) derived from AML patients than that from healthy individuals. The low expression of MYCT1 in AML BM may be due to the hypermethylation in its promoter. MYCT1 expression was strongly associated with French–American–British classifications of AML. The low expression level of MYCT1 was more often observed in patients of M1, M5 and M6 types. *In vitro*, lentiviral particles carrying the complete CDS of MYCT1 gene were used to mediate the forced overexpression of MYCT1 in two AML cell lines, HL-60 and KG-1a. MYCT1 overexpression significantly inhibited cell proliferation, arrested cell cycle at G_0_/G_1_ phase, and downregulated the expression of cyclins D and E. Moreover, MYCT1 overexpression triggered apoptosis in AML cells, which was accompanied by enhanced cleavage of caspase-3 and -9, upregulated expression of B-cell lymphoma 2 (Bcl-2)-associated X protein (Bax), and downregulated Bcl-2. Finally, in BALB/c nude mice bearing xenograft tumors generated by HL-60 and KG-1a cells, we noted that the intratumoral injection of MYCT1 lentivirus repressed tumor growth and led to massive apoptosis. In summary, our results reveal that MYCT1’s promoter is hypermethylated and its expression is downregulated in the BM of AML patients. MYCT1 plays a tumor-suppressive role, and it may serve as a promising target for the genetic therapeutic strategy in treating AML.

## Introduction

Acute myeloid leukemia (AML) is a heterogeneous clonal disorder characterized by the increased proliferation and survival of immature myeloid cells. Current treatment approaches have improved only modestly for the past three decades, and the 5-year survival rate is still unsatisfactory (only 38% for AML patients younger than age 60 years-old) (Burnett et al., 2010; Jemal et al., 2011). Since chemotherapy resistance and relapse are still the major challenges in treating AML (Estey and Dohner, 2006), a better understanding of the molecular mechanisms underlying the tumorigenesis of AML is critical for exploring novel treatment protocols and disease-specific targets, thereby improving the survival of AML patients. Although a variety of gene abnormalities have been found to be associated with AML carcinogenesis (Osato et al., 2001; Bernasconi et al., 2004; Haferlach et al., 2004; Li et al., 2017), the therapeutic potentials of these genes are not fully evaluated, and novel therapeutic targets remain to be investigated.

MYC target 1 (MYCT1), also known as MTLC, is a direct target gene of c-Myc, and it has been first cloned from laryngeal squamous cell carcinoma (LSCC) (Qiu et al., 2003). MYCT1 is located in 6q25, a chromosome region involved in many kinds of cancers, and it has been identified to contain a nuclear location signal motif (Qiu et al., 2003). As a novel candidate of tumor suppressor genes, MYCT1 has previously been found to be downregulated in gastric carcinoma (GC) and it plays an important role in regulating apoptosis and cell cycle process of GC cells (Qiu et al., 2003). Fu et al. (2011) also found that MYCT1 was expressed at a low level in LSCC. These earlier studies suggest an association of MYCT1 downregulation with carcinogenesis. However, the role of MYCT1 in AML is unclear, and whether MYCT1 can serve as a therapeutic target for AML remains to be addressed.

In this study, we first analyzed the promoter methylation and expression levels of MYCT1 in the Bone marrow (BM) derived from both AML patients and healthy individuals. Further, the proliferation, cell cycle and apoptosis of AML cells overexpressing MYCT1 were assessed. Our results revealed that the promoter of MYCT1 gene was hypermethylated and its expression was downregulated in AML BM. Furthermore, MYCT1 overexpression inhibited growth and triggered apoptosis in AML cells *in vitro* and *in vivo*.

## Materials and Methods

### Clinical Samples

This study was approved by the Medical Ethics Committee of Shengjing Hospital of China Medical University. The diagnosis and classification of the AML patients were based on the revised 2008 World Health Organization (WHO) criteria. All participants provided written informed consents, and the whole experiments were in accordance with the Declaration of Helsinki. BM biopsy samples were obtained from 50 AML patients (AML group) at the time of initial diagnosis, prior to chemotherapy. BM samples were also obtained from 50 healthy participants, whose gender and age were matched with the AML patients (Healthy controls group). The healthy individuals were the relatives of leukemia patients who came to our hospital as the potential BM donors.

### Cell Culture

Acute myeloid leukemia cell line HL-60 was purchased from Shanghai Zhong Qiao Xin Zhou Biotechnology Co., Ltd. (Shanghai, China). KG-1a cells were purchased from Procell Life Science & Technology Co., Ltd. (Wuhan, Hubei, China). These two cell lines were authenticated by Procell Life Science & Technology in 2017 (HL-60) and in 2016 (KG-1a), and showed 100% similarity in short tandem repeats (STR) profile when compared with the ATCC database. Cells were cultured in Roswell Park Memorial Institute (RPMI) 1640 medium (Gibco, Grand Island, NY, United States) containing 10% fetal bovine serum (FBS, Hyclone, Logan, UT, United States), 100 U penicillin and 100 mg/ml streptomycin (Gibco^@^) at 37°C in a humidified atmosphere of 5% CO_2_.

### Lentivirus Infection

The lentiviruses were purchased from Gene Pharma (Shanghai, China). HL-Lv-NC and KG-Lv-NC cells were infected with negative control lentiviral particles, while HL-Lv-MYCT1 and KG-Lv-MYCT1 cells were infected with lentiviral particles overexpressing MYCT1. Uninfected HL-60 and KG-1a cells were the parental cells.

For lentivirus infection, HL-60 and KG-1a cells (4 × 10^5^) were grown in 6-well plates for 24 h. The lentivirus was first diluted in RPMI 1640 medium (serum and antibiotic free), and then added into cell culture to infect AML cells. Twenty-four hours later, culture medium was discarded, and cells were cultured in RPMI 1640 medium with 10% FBS.

### Real-Time PCR (RT-PCR)

The mRNA levels of MYCT1 in HL-60 and KG-1a cells were determined by RT-PCR. Total RNA was extracted using a high purity total RNA extraction kit (BioTeke Ltd., Beijing, China) according to the manufacturer’s instructions. cDNAs were synthesized from RNA templates by Super M-MLV reverse transcriptase (BioTeke). The reverse transcription products were then used as templates in SYBR Green reaction mix-mediated RT-PCR analysis (Solarbio, Beijing, China). Primer sequences were as following: MYCT1: 5′-CAGTCTCACCTTCCAGCGA-3′ (forward) and 5′-ACCAGTAGTCAGGACGGCTC-3′ (reverse); GAPDH: 5′-GAAGGTCGGAGTCAACGGAT-3′ (forward) and 5′-CCTGGAAGATGGTGATGGGAT-3′ (reverse). The reaction conditions of reverse transcription were as follows: 25°C for 10 min, 42°C for 50 min, and 95°C 5 min. The PCR amplification conditions were: 94°C for 10 min, 40 cycles of 94°C for 10 s, 60°C for 20 s and 72 °C for 30 s, finally followed by 72°C for 2 min 30 s, 40°C for 5 min 30 s, melting 60°C to 94°C, every 1.0°C for 1 s and 25°C for 1 min. PCR reaction was performed on Exicycler 96 Thermal Real-Time Quantitative Thermal Block (Bioneer, Daejeon, Korea), and GAPDH was used as the internal control. Data were determined via the 2^-ΔΔCT^ method.

### Western Blot Analysis

Anti-MYCT1 antibody (dilution rate 1:1000) was purchased from Proteintech Group Inc. (Wuhan, Hubei, China), anti-cyclin D1 (dilution rate 1:500) antibody was purchased from Boster Biological Technology Co., Ltd. (Pleasanton, CA, United States), anti-cyclin E (dilution rate 1:1000) and anti-cleaved caspase-3 (dilution rate 1:1000) antibodies were purchased from Abcam (Cambridge, MA, United States), anti-B-cell lymphoma 2 (anti-Bcl-2, dilution rate 1:1000) and anti-Bcl-2-associated X protein (anti-Bax, dilution rate 1:1000) antibodies were purchased from Sangon Biotech (Shanghai, China), anti-cleaved caspase-9 antibody (dilution rate 1:1000) was purchased from CST Inc. (Danvers, MA, United States) and anti-GAPDH antibody (dilution rate 1:500) was purchased from Bioss Inc. (Woburn, MA, United States). For western blot analyses, proteins were extracted using a total protein extraction kit (Beyotime Bio., Shanghai, China), and protein concentration was determined using a BCA Protein Assay Kit (Beyotime) following the manufacturer’s instructions. Proteins (20–40 μg) were separated by 12% sodium dodecyl sulfate–polyacrylamide gel electrophoresis (SDS-PAGE) and transferred to polyvinylidene difluoride (PVDF) membranes (Millipore Co, Billerica, MA, United States). Then the PVDF membranes were probed with one of the above primary antibodies at 4°C overnight, followed by incubation with a HRP-conjugated goat anti-rabbit IgG or a HRP-conjugated donkey anti-goat IgG secondary antibody (Beyotime) for 1 h at room temperature. The relative intensities of protein bands were visualized by ECL system (Beyotime) and normalized to GAPDH. Quantitative analysis was performed by Gel-Pro-Analyzer software.

### Bisulfite Modification and Bisulfite-Specific PCR (BSP)

Genomic DNA was randomly selected from nine AML patients and nine healthy individuals for methylation status screening. Approximately 500 ng of DNA sample was bisulfate-modified using the EZ DNA Methylation-Gold Kit^TM^ (Zymo Research, Orange, CA, United States) according to the manufacturer’s instructions. Based on the functional promoter sequence of MYCT1 gene, the primers were used as follows: 5′-ATGGTGGAATTTTATTTTTAGTAAAA-3′ (forward) and 5′-ACTCACTACAACCTACCCCTC-3′ (reverse) in BSP detection. The PCR reaction was performed in a 20-μl reaction system, starting with denaturation at 95°C for 5 min, then 30 cycles of denaturation at 95°C for 10 s, annealing at 52°C for 20 s, extension at 72°C for 30 s, followed by an extra extension at 4°C for 5 min. The amplified DNA fragments were mixed with Goldview (Solarbio, Beijing, China) loading dye and separated by electrophoresis in 1.5% agarose gel. After that, the gel was photographed by using Gel Imaging System (Beijing Liuyi Biotechnology, Beijing, China) and the fragments were sequenced by BiQ Analyzer 2.0.

### Cell Counting Kit-8 (CCK-8) Assay

Cells were harvested and seeded in 96-well plates (5 × 10^3^ cells per well) and incubated at 37°C for lentivirus infection. Forty-eight hours later, cell viability was determined at 24, 48, 72 or 96 h. CCK-8 working solution (Beyotime) was added (10 μl per well) and cells were incubated for 1 h at 37°C. Then, the absorbance of each well was read on a microplate reader (BIOTEK, Winooski, VT, United States) at 450 nm. OD value was used for analysis.

### Apoptosis and Cell Cycle Assays

For apoptosis assay, cells were stained with an Apoptosis Assay Kit (Wanlei Bio, Shenyang, China) according to the manufacturer’s instructions, and then detected by fluorescence-activated cell sorting (FACS) analysis. Briefly, cells were seeded in 6-well plates for lentivirus infection. Forty-eight hours later, cells were harvested and washed twice with cold phosphate buffered saline (PBS; double-helix, Shanghai, China). Subsequently, cells were incubated with 5 μl Annexin V-Light 650 and 10 μl propidium iodide (PI) in the dark for 15 min at room temperature. The apoptotic cells were detected by FACS analysis (Accuri C6, BD Biosciences) and the data were analyzed using BD Accuri C6 Software (BD Biosciences) on 10,000 events.

For cell cycle assay, cells were labeled with PI using a Cell Cycle Assay Kit (Beyotime) according to the manufacturer’s procedures. Briefly, 48 h after lentivirus infection, cells were harvested, washed twice with cold PBS, and fixed with ice-cold 70% ethanol for 2 h. Fixed cells were subsequently treated with 25 μl PI. Finally, 10 μl ribonuclease (RNase A) was added to the cells. The DNA content was then quantitated by FACS Accuri C6 (BD Biosciences), with an excitation wavelength of 488 nm and an emission wavelength of 625 nm. Data were analyzed using BD Accuri C6 Software (BD Biosciences) on 10,000 events.

### Hoechst Staining

For Hoechst staining, 48 h post-lentivirus infection, cells were harvested and fixed with 0.5 ml fixing solution for 20 min at room temperature. After washing with PBS, cells were stained with Hoechst staining kit (Beyotime) for 5 min according to the manufacturer’s instructions. The slides were observed under a fluorescence microscope (OLUMPUS, Japan). Images were taken at 400× magnification.

### Xenograft Tumor Model

All animal experiments were conducted with the approval by Medical Ethics Committee of Shengjing Hospital of China Medical University, and all procedures conformed to the Guide for the Care and Use of Laboratory Animals.

Healthy female BALB/c-nu nude mice (18∼20 g) aged 2 months were purchased from Huafukang Bio Co., Inc. (Beijing, China) and raised in our animal center under standard laboratory conditions.

Thirty-six mice were randomly divided into six groups (six mice per group). Group 1: HL-60 group, mice were first subcutaneously inoculated with HL-60 cells and then received intratumoral injections of PBS; Group 2: HL-Lv-NC group, mice were first subcutaneously inoculated with HL-60 cells and then received intratumoral injections of HL-Lv-NC; and Group 3: HL-Lv-MYCT1 group: mice were first subcutaneously inoculated with HL-60 cells and then received intratumoral injections of HL-Lv-MYCT1. The above experiments were also performed in KG-1a formed xenograft tumor (Groups 4–6).

Briefly, HL-60 or KG-1a cells (1 × 10^7^/0.1 ml serum-free medium) were implanted subcutaneously into the right armpit of a BALB/c-nu mouse. Seven days later, the majority of tumors grew to about 100 mm^3^. Then, a total of 2 × 10^9^ TU Lv-NC or Lv-MYCT1 lentiviral particles were injected into the tumors at day 7, 12 and 17, respectively. The tumor volume (mm^3^) was estimated every 3 days. After 21 days of implantation, the mice were euthanized. After measuring tumor weights, the tumors were fixed in 10% buffered formalin for further examinations.

### Hematoxylin-Eosin Staining (HE Staining)

The fixed tumor tissues were immersed in dimethylbenzene (Sinopharm Group Ltd., Shanghai, China) for 20 min and then in paraffin (Sinopharm Group Ltd., Shanghai, China) at 60°C for 2 h. Paraffin blocks were cut into 5-μm slices using a microtome and deparaffinized. Then, the slices were stained with hematoxylin (Solarbio) and eosin (Sinopharm group) for histological examination. The sections were examined by a light microscope and were photographed (OLUMPUS). Images were taken at 400× magnification.

### Terminal-Deoxynucleoitidyl Transferase Mediated Nick End Labeling (TUNEL) Assay

The tumor sections were deparaffinized with 0.1% Triton X-100 (Beyotime) prior to blocking with 3% H_2_O_2_ (Sinopharm Group). Ten minutes later, the sections were incubated with TUNEL reaction medium (Roche, Basel, Switzerland) at 37°C for 1 h away from light. After the reaction was stopped, the sections were washed three times with PBS. Thereafter, the sections were re-stained with hematoxylin (Solarbio) for 3 min, washed with running water, and then observed under a light microscope (OLUMPUS). Cells with fragmented DNA could be stained brown, and were considered as apoptotic cells. All images were taken at 400× magnification.

### Statistical Analysis

All experiments were repeated at least three times unless otherwise noted. Data were presented as mean ± standard deviation, and analyzed via one-way analysis of variance (ANOVA) followed by Bonferroni’s multiple comparison. Data analysis and plotting were conducted in GraphPad Prism 4.0 (GraphPad software, San Diego, CA, United States). Clinical data were analyzed via SPSS 22.0 software (Chicago, IL, United States). The association between MYCT1 expression and age/gender was analyzed via Pearson’s chi-square test. The association between MYCT1 expression and French–American–British (FAB) category was analyzed via likelihood ratio chi-square test (this test was used when there were groups with count less than five). A *p*-value < 0.05 was considered significant.

## Results

### MYCT1 Promoter Is Hypermethylated and Its Expression Is Lower in BM of AML Patients

**FIGURE 1:**
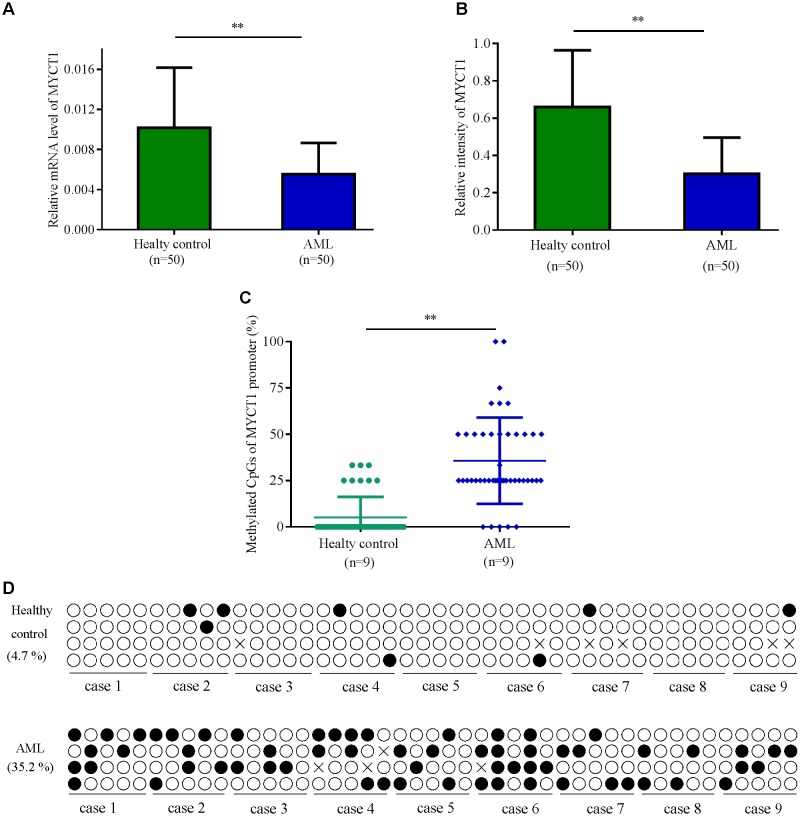
The expression levels of MYCT1 in BM of AML patients and Healthy controls. Relative mRNA **(A)** and protein **(B)** levels of MYCT1 in the BM of AML patients and healthy controls were tested by RT-PCR and Western blot analysis, respectively (*n* = 50). **(C)** Methylation density of MYCT1 gene in AML patients and Healthy controls as analyzed by BSP (*n* = 9); **(D)** Methylation status of the specific promoter region of the MYCT1 gene. Each line of circles indicated the sequence of an individual clone; ∘ represented an unmethylated CpG site and ∙ represented a methylated CpG site. ^∗∗^*P* < 0.01 versus healthy group and BM, bone marrow.

Low expression of MYCT1 has been found in the tumor tissues of gastric carcinoma patients (Qiu et al., 2003). To explore the expression level of MYCT1 in AML, BM was collected from 50 Chinese AML patients and 50 healthy individuals. The mRNA and protein levels of MYCT1 in both AML group and health group were determined by RT-PCR and Western blot analysis, respectively. Data showed that both the mRNA and protein levels of MYCT1 were downregulated in AML BM (*P* < 0.01 versus normal BM, **Figures 1A,B**). BSP was performed to detect the methylation status of MYCT1 promoter in BM. Our results showed that MYCT1 was hypermethylated in AML BM as compared with the normal BM. The methylation density (proportion of methylated cytosine-guanine pair (CpG) sites within a specific promoter region) of the MYCT1 gene was significantly increased in the AML BM (*P* < 0.01 versus normal BM, **Figures 1C,D**).

Next, to explore the correlation between MYCT1 expression and AML clinical characteristics, AML patients were divided into two groups: the low MYCT1 group (*n* = 25, fold-change ≥ median), and the high MYCT1 group (*n* = 25, fold-change ≤ median). MYCT1 expression was not associated with age (*p* = 0.396) or gender (*p* = 0.569) in AML patients (**Table 1**). Further, we found that MYCT1 expression was strongly associated with FAB category (*p* = 0.03), a hematopathologic criteria for the classification of AML (Burns et al., 1981). The low expression level of MYCT1 was more often observed in patients with M1, M5, and M6 (**Table 1**).

**Table 1:** Correlation between MYCT1 expression and clinical characteristics of AML patients (*n* = 50).

Characteristics		MYCT1		*P*-value
		Low	High	
Age	>46	14	11	0.396
	<46	11	14	
Gender	Male	13	15	0.569
	Female	12	10	
FAB category	M0	0	2	0.030^∗^
	M1	2	0	
	M2	3	9	
	M3	5	6	
	M4	4	4	
	M5	10	3	
	M6	1	0	
	M7	0	1	

*P < 0.05, statistically significant. Data of age and gender were analyzed via Pearson Chi square test, while data of FAB classification were analyzed with likelihood ratio chi square test*.

### Overexpression of MYCT1 in AML HL-60 and KG-1a Cells by Lentiviral Infection

**FIGURE 2:**
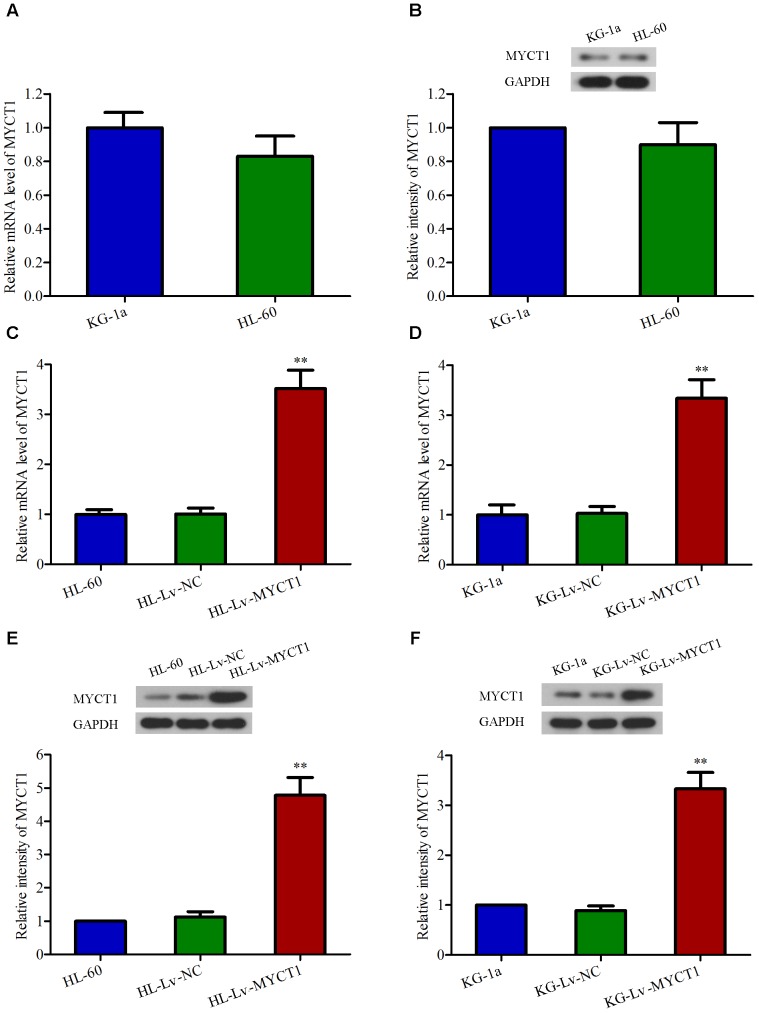
Overexpression of MYCT1 in HL-60 and KG-1a AML cells by lentiviral infection. The mRNA **(A)** and protein **(B)** levels of MYCT1 in HL-60 and KG-1a cell lines were examined by RT-PCR and Western blot analysis, respectively. **(C–F)** HL-60 and KG-1a cells were infected with negative control lentiviral particles (Lv-NC) or lentiviral particles overexpressing MYCT1 (Lv-MYCT1). The mRNA **(C,D)** and protein **(E,F)** levels of MYCT1 in HL-60 **(C,E)** and KG-1a **(D,F)** cells were assessed by RT-PCR and Western blot analysis, respectively. ^∗∗^*P* < 0.01 versus HL-Lv-NC or KG-Lv-NC cells.

It has been documented that MYCT1 can suppress cell growth and induce apoptosis in LSCC and gastric carcinoma cells (Yang et al., 2012). Here we speculate that MYCT1 may act as a tumor suppressor in AML carcinogenesis. Firstly, the mRNA and protein levels of MYCT1 in HL-60 and KG-1a cell lines were examined by RT-PCR and Western blot analysis, respectively. HL-60 and KG-1a cells were identified as MYCT1-postitive cells (**Figures 2A,B**). Then, to study the role of MYCT1, HL-60, and KG-1a cells were infected with MYCT1-overexpressed lentivirus (Lv-MYCT1) or empty lentivirus (Lv-NC). Lv-MYCT1 significantly increased MYCT1 mRNA and protein expression in both HL-60 and KG-1a cells, but Lv-NC did not (*P* < 0.05 versus Lv-NC; **Figures 2C–F**).

### Overexpression of MYCT1 Inhibits Cell Proliferation and Induces Cell Cycle Arrest in AML Cells

**FIGURE 3:**
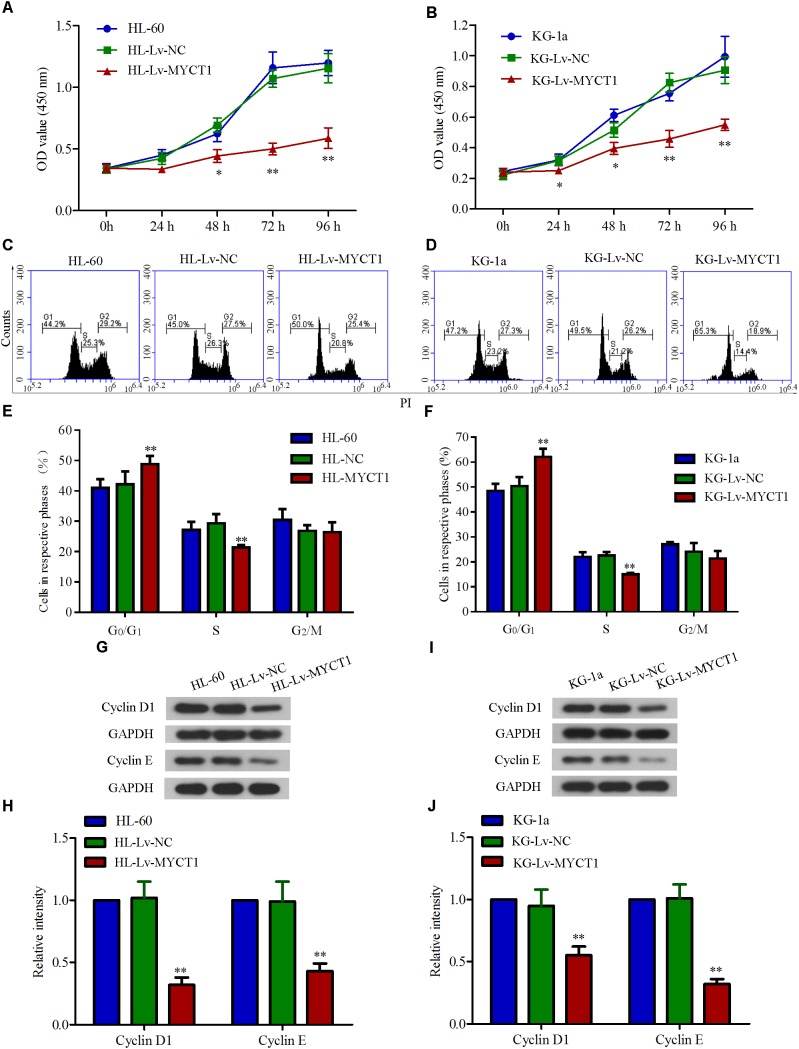
Overexpression of MYCT1 inhibits cell proliferation and induces cell cycle arrest in HL-60 and KG-1a cells. Cells proliferation was measured by CCK-8 assay in HL-60 **(A)** and KG-1a **(B)** cells at 24 h, 48 h, 72 h and 96 h after lentiviral delivery. **(C–F)** MYCT1 overexpression-induced cell cycle arrest in HL-60 and KG-1a cells. Changes in cell cycle distribution of MYCT1-overexpressing HL-60 **(C)** and KG-1a **(D)** cells were determined by FACS analysis, and the proportions of cells in G_0_/G_1_, S and G_2_/M phase were calculated **(D,F)**. **(G–J)** The expression levels of cell cycle regulatory proteins in HL-60 **(G,H)** and KG-1a **(I,J)** cells were examined by Western blot analysis. ^∗^*P* < 0.05 and ^∗∗^*P* < 0.01 versus HL-Lv-NC or KG-Lv-NC cells.

MYCT1 has been demonstrated to play an important role in regulating cell proliferation as well as cell cycle (Yin et al., 2002). Hence, the effects of MYCT1 overexpression on AML cell proliferation and cell cycle were investigated in HL-60 and KG-1a AML cells. Cell proliferation over a time course (0, 24, 48, 72, and 96 h) was assessed by CCK-8 assay, and the data showed a significant reduced proliferation in cells overexpressing MYCT1 (*P* < 0.01 versus Lv-NC, **Figures 3A,B**). Further, overexpression of MYCT1 resulted in a marked accumulation of cells in G_0_/G_1_ phase as demonstrated by cell cycle analysis (*P* < 0.01 versus Lv-NC, **Figures 3C–F**).

To further study the mechanisms underlying MYCT1 overexpression-induced cell cycle arrest in AML cells, the expression levels of cell cycle-related proteins, cyclin D1 and cyclin E were determined by Western blot analysis. As shown in **Figures 3G–J**, the expression levels of both cyclin D1 and cyclin E were downregulated in response to MYCT1 overexpression (*P* < 0.01 versus Lv-NC). These results suggested that MYCT1 overexpression arrested AML cells at G_0_/G_1_ phase at least by downregulating cyclins D1 and E.

### Overexpression of MYCT1 Induces Apoptosis in AML Cells

**FIGURE 4:**
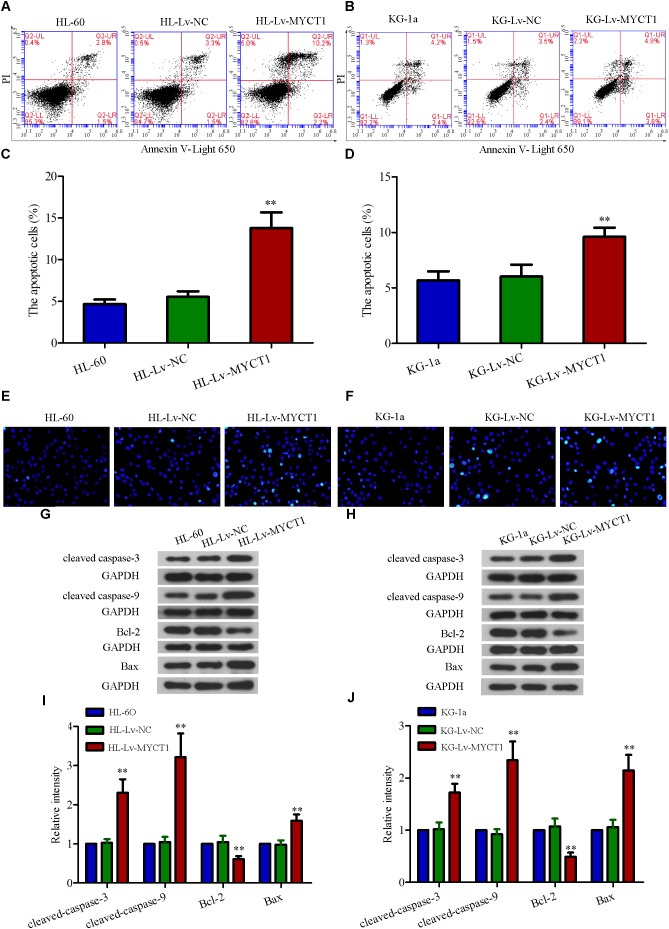
Overexpression of MYCT1 induces apoptosis in HL-60 and KG-1a cells. **(A,B)** The apoptotic cells were detected by FACS analysis after staining with PI/Annexin V-Light 650, and the apoptosis rates were shown in parts **(C,D)** (apoptotic cell fraction = UR + LR). UR quadrant represents Annexin V and PI positive staining cells (apoptotic cells), while LR quadrant represents Annexin V positive and PI negative staining cells (early apoptotic cells). **(E,F)** Apoptosis, as induced by overexpression of MYCT1 in HL-60 **(E)** and KG-1a **(F)** cells, was determined by Hoechst staining. ^∗∗^*P* < 0.01 versus HL-Lv-NC or KG-Lv-NC cells. Scale bar: 50 μm. **(G,H)** The levels of apoptosis-related proteins, including cleaved caspase-3, cleaved caspase-9, Bcl-2 and Bax were assessed by Western blot analysis. **(I,J)** Quantitative analysis of the gray intensity values. ^∗∗^*P* < 0.01 versus HL-Lv-NC or KG-Lv-NC cells. UR, Upper right and LR, Lower right.

Next, we explored how MYCT1 overexpression affected AML cell apoptosis by performing annexin V-PI double staining analysis. As shown in **Figures 4A,B**, the apoptotic cells were robustly increased after MYCT1 overexpression (*P* < 0.01 versus Lv-NC, **Figures 4C,D**). In addition, Hoechst assay was performed to examine DNA fragmentation, another effective indicator of apoptosis (**Figures 4E,F**). It was found that the fraction of Hoechst-positive cells was significantly increased in HL-Lv-MYCT1 and KG-Lv-MYCT1 cells, supporting annexin V-PI results. Moreover, the expression levels of a group of critical apoptosis markers were detected by Western blot analysis (**Figures 4G–J**). As expected, the levels of caspase cascade proteins, such as cleaved caspase-3 and -9, were elevated in HL-60 and KG-1a cells after MYCT1 overexpression (*P* < 0.01 versus Lv-NC). Activation of endogenous apoptotic pathway is regulated by the balance between anti-apoptotic proteins such as Bcl-2, and pro-apoptotic proteins such as Bax. Thus, the expression levels of Bcl-2 and Bax were also examined by Western blot analysis. It was found that the expression of Bcl-2 was downregulated, whereas the expression of Bax was upregulated after MYCT1 overexpression (*P* < 0.01 versus Lv-NC). These results suggested that the downregulated Bcl-2 and the upregulated Bax were responsible for the activation of endogenous apoptotic pathway in AML cells overexpressing MYCT1.

### Intratumoral Injection of Lv-MYCT1 Inhibits Xenograft Tumor Growth and Induces Apoptosis

**FIGURE 5:**
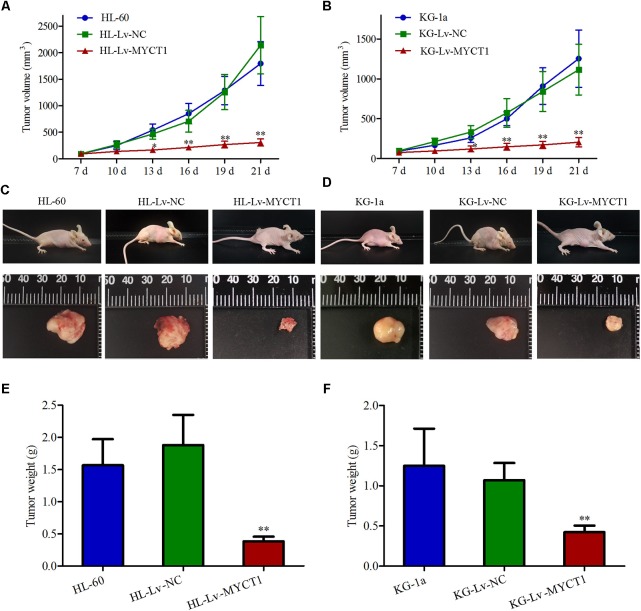
Overexpression of MYCT1 delays the growth of AML xenograft tumors *in vivo*. HL-60 and KG-1a cells were first subcutaneously implanted into the flanks of BALB/c-nu mice. Mice were given intratumoral injections of Lv-NC or Lv-MYCT1 at day 7, 12, and 17. **(A,B)** Tumor volumes of AML xenografts in different groups. **(C,D)** Photographs of the mice bearing AML xenograft tumors **(C,D**, upper panel**)** and the isolated tumors **(C,D**, lower panel**)**. **(E,F)** Tumor weights of AML xenografts in different groups. ^∗^*P* < 0.05 and ^∗∗^*P* < 0.01 versus HL-Lv-NC or KG-Lv-NC cells.

Our *in vitro* experiments revealed the anti-proliferative and pro-apoptotic effects of MYCT1 in AML cells (**Figures 3**, **4**). To further investigate the effect of MYCT1 overexpression on the growth of AML cells *in vivo*, a xenograft tumor model was established in BALB/c-nu mice. We found that intratumoral injection of Lv-MYCT1 significantly delayed the growth and reduced the size of subcutaneous xenograft tumors (**Figures 5A–D**). Twenty-one days after tumor cell inoculum, marked reduction in tumor weight was induced by MYCT1 overexpression (*P* < 0.01 versus Lv-NC, **Figures 5E,F**).

**FIGURE 6:**
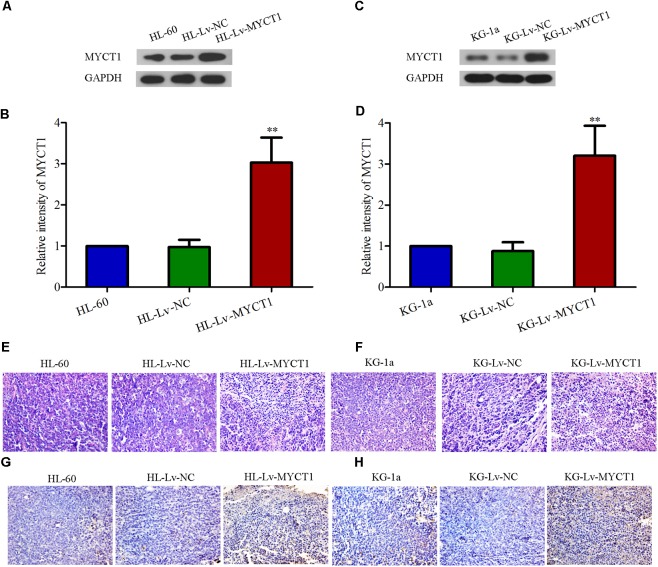
Overexpression of MYCT1 induces tumor-cell apoptosis in a mouse xenograft model *in vivo*. **(A–D)** The expression levels of MYCT1 in AML xenograft tumors determined by Western blot analysis. **(E,F)** Histological examination of xenograft tumors in different groups by HE staining. Scale bar: 50 μm. **(G,H)** Apoptosis in the xenograft tumors was determined by TUNEL assay. Scale bar: 50 μm and ^∗∗^*P* < 0.01 versus HL-Lv-NC or KG-Lv-NC cells.

Western blot results showed that the MYCT1 expression was significantly increased in the tumor injected with Lv-MYCT1 (*P* < 0.01 versus Lv-NC, **Figures 6A–D**). Moreover, as illustrated in **Figures 6E,F**, Lv-NC did not induce significant morphological changes in the xenograft tumor, whereas MYCT1 overexpression promoted tumor cell apoptosis: cytoplasm lysis and nuclear condensation. The apoptosis was further confirmed with TUNEL assay (**Figures 6G,H**). These results showed that MYCT1 inhibited tumor growth, and promoted apoptosis in AML xenografts in mice.

## Discussion

Various genetic and epigenetic alterations in oncogenes and tumor suppressor genes play an important role in leukemogenesis (Mullighan et al., 2011; Andrade et al., 2014; Li and Zhu, 2014), and identification of novel genes linked to AML pathogenesis is needed. Previous work is focused on the relationship between AML with oncogenes, such as Bcl-2 (Pan et al., 2017) and EGFR (Mahmud et al., 2016), with tumor suppressor genes, such as p53 (Pan et al., 2017), and with Bcl-2 homology domain 3 (BH3)-only proteins, such as Puma, Bim, and Noxa (Bilardi et al., 2016; Grundy et al., 2018). There is no clear conclusion whether AML carcinogenesis is related with the putative tumor suppressor gene MYCT1. Our study shows that MYCT1 promoter is hypermethylated and its expression is downregulated in AML BM. MYCT1 overexpression contributes to the abnormal growth and apoptosis of HL-60 and KG-1a cells *in vitro* and *in vivo*.

MYCT1 is the target gene of c-Myc, and its closest homolog, mouse c-Myc target in myeloid cells-1 (MT-MC1) has been found to recapitulate global c-Myc-induced phenotypes in animal model (Rothermund et al., 2005). MT-MC1 can regulate cell proliferation, cell cycle progression, apoptosis, differentiation, and genomic stability (Yin et al., 2002). Therefore, like MT-MC1, MYCT1 may also participate in regulating these processes. Recently, another novel MYCT1 transcript, MYCT1-TV has been found to be regulated by c-Myc and may participate in laryngeal carcinogenesis (Fu et al., 2011). All these studies imply that MYCT1 may have a role in regulating carcinogenesis. In LSCC, Yang et al. (2012) reported that c-Myc regulated MYCT1 transcription and that DNA methylation in MYCT1 promoter interfered its binding to c-Myc. In this study, we found that MYCT1 promoter was hypermethylated in AML BM, which may explain the corresponding low expression of MYCT1.

French–American–British classification is a core feature of the large WHO category of AML, and it has been further optimized by combining the analysis of morphology, immunophenotyping, cytogenetics, and molecular genetics of acute leukemia cells (Leonard et al., 2017). Aberrant gene expression may be associated with AML FAB classification. For instance, Somerville et al. (2018) reported that the expression of Iroquois homeodomain transcription factor IRX3 is strongly correlated with reduced myelomonocytic differentiation. We also analyzed the correlation between MYCT1 expression and AML classification and found that MYCT1 expression was strongly associated with FAB AML category. The low expression level of MYCT1 was more often observed in patients of M1, M5, and M6 types, but not in other FAB AML types. Although these data suggest MYCT1 as a potential prognosis maker in AML classification, further analysis of the association between MYCT1 expression and AML classification in a larger scale of clinical samples is needed.

Results from the *in vitro* and animal study showed that MYCT1 overexpression led to proliferation inhibition and cell cycle arrest in HL-60 and KG-1a cells. c-Myc is suggested as a proto-oncogene, and thus its downregulation is thought to inhibit cancer cell survival. As MYCT1 overexpression could inhibit AML cell growth, c-Myc was anticipated to be downregulated. Unexpectedly, we found that the expression of c-Myc was slightly upregulated in HL-60 and KG-1a cells overexpressing MYCT1 (data not shown). These results suggested that the anti-tumor effects of MYCT1 in AML cells may be not affected by c-Myc. More effort is needed to elucidate the underlying mechanisms.

It is well-documented that cell cycle is an essential process of cell proliferation, which plays an key role in tumorigenesis (Sherr, 1996). Liddiard et al. (2012) reported that overexpression of MYCT1 reduced the viability of AML cells under serum free medium without elucidating the underlying mechanisms. Cyclins include cyclin A, B, D, E, G, and H, and work with cyclin-dependent protein kinases (CDKs) to regulate the cell cycle progression (Hunt, 1989; Lee and Yang, 2003; Sanchez and Dynlacht, 2005). Cyclin D1 and cyclin E are the key players in the late G1 phase. Cyclin D and CDK4 constitute complexes that phosphorylate Rb and cause Rb dissociation from E2F. Subsequently, E2F-dependent transcription is initiated to facilitate DNA replication (Spruck et al., 1999) and G1-to-S phase transition (Resnitzky and Reed, 1995; Massague, 2004). Cyclin D1 is frequently overexpressed in multiple cancers, leading to accelerated tumor progression with shortened G1 phase (Simpson et al., 2001; Gautschi et al., 2007). Herein, we found that MYCT1 overexpression arrested AML cells at G1 phase. Given to the important regulatory role of cyclins, it is plausible that the cell cycle arrest of AML cells overexpressing MYCT1 may be associated with the downregulation of cyclins D1 and E.

Cell death occurs through various processes, including autophagy, apoptosis, and necrosis in malignancies, with apoptosis as the best described process in AML (Sachs, 1993; Sachs and Lotem, 1993; Sinkovics and Horvath, 1994). Apoptosis is mediated through the caspase cascade (Adams, 2003). The decision of endogenous apoptosis mediated by caspase-9 is made by the interaction between pro-apoptotic proteins, such as Bax, and anti-apoptotic proteins, such as Bcl-2 (Adams and Cory, 2007). Specifically, the ratio of pro-apoptotic Bax and anti-apoptotic Bcl-2 determines the response to an endogenous apoptotic signal (Pawlowski and Kraft, 2000). Bcl-2 binds to Bax to prevent its activation. Both Bax and Bcl-2 have been confirmed to play critical roles in the leukemogenesis as well as multi-drug resistance of AML cells (Soderquist and Eastman, 2016; Pan et al., 2017; Reyna et al., 2017). The released Bax facilitates the formation of outer-mitochondrial membrane spanning pores, which triggers the activation of the caspase cascade orchestrated by caspase-3. Herein, we observed that overexpression of MYCT1 induced apoptosis in HL-60 and KG-1a cells, and upregulated Bax, downregulated Bcl-2, and enhanced cleavage of caspase-3 and -9. Similar proapoptotic role of MYCT1 was also found in the AML cell xenografts. These results suggest that MYCT1 affects AML cell apoptosis by modulating the endogenous apoptotic pathways.

## Conclusion

In conclusion, our study demonstrates that the expression of MYCT1 is reduced in AML BM, which may be associated with the hypermethylation in its promoter. Overexpression of MYCT1 by lentivirus infection significantly suppresses proliferation and induces apoptosis of AML cells both *in vitro* and *in vivo*. Our findings suggest MYCT1 as a potential therapeutic target for the treatment of AML.

## Ethics Statement

Ethics approval was obtained from Medical Ethics Committee of Shengjing Hospital of China Medical University and written informed consent was obtained from each patient prior to sample collection.

## Author Contributions

SF and JZ designed the experiments. SF, YF, FC, YH, and BQ performed the experiments. SF and YF analyzed the data. YH and BQ contributed to the preparation of reagents, materials, and analysis tools. SF and JZ wrote the manuscript. All authors read and approved the final manuscript.

## Conflict of Interest Statement

The authors declare that the research was conducted in the absence of any commercial or financial relationships that could be construed as a potential conflict of interest.
